# Efficacy and acquired resistance for EGFR-TKI plus thoracic SBRT in patients with advanced EGFR-mutant non–small-cell lung cancer: a propensity-matched retrospective study

**DOI:** 10.1186/s12885-021-08228-2

**Published:** 2021-04-30

**Authors:** Xia Wang, Zhimin Zeng, Jing Cai, Peng Xu, Pingan Liang, Yuxi Luo, Anwen Liu

**Affiliations:** 1 Department of Oncology, The Second Affiliated Hospital of Nanchang University, Nanchang University, No.1 Minde Street, Nanchang, 330000 Jiangxi Province People’s Republic of China; 2 Jiangxi Key Laboratory of Clinical Translational Cancer Research, Nanchang, Jiangxi Province 330000 People’s Republic of China

**Keywords:** Non-small-cell lung cancer, Epidermal growth factor receptor tyrosine kinase inhibitors, Stereotactic body radiation therapy, Propensity score matching, Acquired resistance mechanism

## Abstract

**Background:**

This retrospective study aimed to evaluate the efficacy of epidermal growth factor receptor (EGFR) tyrosine kinase inhibitors (TKIs) with stereotactic body radiation therapy (SBRT) and to elucidate potential mechanisms of acquired resistance.

**Methods:**

Patients with advanced NSCLC harboring positive EGFR mutations after initial TKI therapy for at least 8 weeks were eligible for SBRT between August 2016 and August 2019. Eligible patients were treated with thoracic SBRT, and TKI was continued after SBRT until it was considered ineffective. The control group was treated with TKIs monotherapy. Propensity score matching (PSM, ratio of 1:2) was used to account for differences in baseline characteristics. Overall survival (OS), progression-free survival (PFS), treatment safety and resistance mechanisms were evaluated.

**Results:**

Three hundred eight patients were included in the study population. Among them, 262 patients received TKIs alone, and 46 patients received TKIs with SBRT. Baseline characteristics were not significantly different between the two cohorts after PSM. The median PFS was 19.4 months in the TKIs +SBRT group compared to 13.7 months in the TKIs group (*p* = 0.034). An influence on OS has not yet been shown (*p* = 0.557). Of the 135 patients evaluated after PSM, 28 and 71 patients in the TKIs and TKIs +SBRT cohorts, respectively, had plasma cell-free DNA (cfDNA) next-generation sequencing (NGS) performed at baseline and disease progression. In the TKIs +SBRT cohort, the NGS results showed that T790M mutations were detected in 64.3% (18/28) of patients. Patients in the TKIs cohort exhibited fewer T790M-positive mutations (40.8%, *p* = 0.035) compared to patients in the TKIs +SBRT cohort.

**Conclusion:**

Real world data prove that TKIs plus thoracic SBRT significantly extend PFS with tolerable toxicity. The mutation ratio of T790M was increased in the TKIs +SBRT group compared to the TKIs only group. Further randomized studies are warranted.

## Background

Lung cancer is the leading cause of cancer-associated death in the world, and non-small-cell lung cancer (NSCLC) accounts for approximately 80% of cases [1, 2]. Approximately 30–40% of patients with NSCLC exhibit epidermal growth factor receptor (EGFR) mutations, and targeted therapy has become the standard treatment for patients with EGFR mutations. A number of studies [3–6] have confirmed that patients with advanced NSCLC with EGFR mutations can gain survival benefits from multiple generation tyrosine kinase inhibitors (TKIs), such as erlotinib, gefitinib or afatinib, and the efficacy is better than systemic chemotherapy. EGFR-TKIs therapy has become the first-line treatment for such patients instead of chemotherapy.

Although the efficacy of EGFR-TKIs is significant, the median progression-free survival (PFS) is only approximately 9–13 months, meaning that TKI resistance invariably develops [7–9]. Several mechanisms of resistance have been identified, of which the T790M mutations account for nearly 50% of acquired resistance mechanisms to EGFR-TKIs [10]. Local consolidation therapy (LCT) might allow patients to continue on the same TKI treatments longer and significantly delays patients from having to switch to other alternative systemic options. Several small prospective trials have shown that LCT, like surgery or stereotactic body radiation therapy (SBRT), can prolong PFS in patients with NSCLC and delay drug resistance in EGFR-TKIs when patients develop oligoprogression, oligometastases or oligopersistence [11, 12]. Our prior study demonstrated that the combination of brain stereotactic radiosurgery and TKIs conveyed increased intracranial PFS and overall survival (OS) compared to TKIs alone in EGFR-mutant lung adenocarcinoma patients [13]. Furthermore, in one pattern of failure analysis of EGFR-mutant patients, almost 50% of recurrences after TKIs therapy occurred first in primary or pre-existing metastatic sites [14]. The lung was the most common site of initial progression, and 45% of patients progressed in primary sites (with or without concurrent metastatic sites) [14].

Therefore, we postulated that thoracic SBRT after initial TKIs therapy could have several benefits, including prevention of lung symptoms from a growing tumor, prevention of secondary seeding by TKI-resistant clones, and enabling continuation with existing TKIs treatments. However, there is limited information about TKIs +SBRT in this setting, and there are no real-world data reported about the mechanisms of acquired resistance to a TKIs +SBRT regimen. Here, we conducted a retrospective study describing a single institution’s experience using SBRT for lung lesions and continuation of TKIs to treat advanced NSCLC patients. We sought to evaluate the PFS benefits for TKIs +SBRT and to illustrate the mechanism underlying acquired resistance using next-generation sequencing (NGS).

## Methods

### Patients

This single center, retrospective study was approved by institutional ethics committee of Second Affiliated Hospital of Nanchang University, Nanchang, China. From the clinical records database of our center, we reviewed a total of 1485 patients diagnosed with NSCLC between August 2016 and August 2019. The eligibility criteria included the following: (1) histological diagnosis of stage III or metastatic/recurrent NSCLC; (2) in the TKIs +SBRT cohort, disease in the lung was limited to 1–3 lesions plus the primary, size 1–5 cm [14–16]; (3) the presence of activating EGFR mutations; (4) age older than 18 years; (5) treatment regimens: TKIs alone or TKIs plus thoracic SBRT. In the 1:2 match, patients who received TKIs plus SBRT were individually matched to two control patients who received TKIs alone. Variables used in the propensity score matching (PSM) included metastatic status and number of lines of TKIs therapy [17]. Patients with initial brain metastases were eligible if they were treated with local treatment (surgery or radiotherapy) and remained clinically stable for at least 8 weeks. Major exclusion criteria included other previous thoracic radiotherapy or prior TKIs therapy. Oligometastatic disease was defined as the presence of ≤5 lesions in 1 to multiple organs at the initiation of TKIs, while poly-metastatic disease was defined as > 5 metastatic lesions [18]. Oligoprogression was defined as being in a polymetastatic state with progressive lesions, while all other lesions were controlled with TKIs. Oligopersistence was defined as stable residual disease sites (in five or fewer sites) after systemic treatment [18, 19].

### Treatment

Forty-six patients with 64 lung lesions treated with at least 8 weeks of TKIs (erlotinib, gefitinib, icotinib, osimertinib or afatinib) followed by SBRT before progression or at the time of thoracic oligoprogression were identified (TKIs plus SBRT group), while 262 patients treated with TKIs were identified (TKIs only group). TKIs were administered for the duration of the SBRT and continued after SBRT until they were considered ineffective or patients developed unacceptable toxicity. SBRT planning was performed using the Monaco planning system and was delivered using the Elekta Versa HD medical linear accelerator. The total dose for SBRT was 70 Gy administered in 10 fractions, 60 Gy administered in 8 fractions, or 50 Gy administered in 5 fractions. Treatment was administered once a day, 5 days per week. Baseline laboratory analyses (hematologic and biochemical profiles) were evaluated every 4 weeks. Magnetic resonance imaging of the brain, chest computed tomography (CT) and upper abdominal CT were performed 1 and 3 months after SBRT and then every 2 months until death or last follow-up.

### Next-generation sequencing

NGS of plasma cell-free DNA (cfDNA) at baseline and progression after TKIs was performed. In the TKIs +SBRT cohort, TKI therapy was continued after SBRT for progressive lesions in the lung, and NGS was performed after further progression. Genomic DNA from whole blood was extracted using the QIAamp DSP Circulating Nucleic Acid Kit (Qiagen, Hilden, Germany) according to the manufacturer’s protocols [20]. The libraries were paired-end sequenced on Illumina NextSeq 500 NGS platforms according to the manufacturer’s instructions [20]. The targeted sequencing depth was > 12,000× for the whole blood control samples.

### Statistical analysis

PFS was defined from the start date of TKIs therapy to first disease progression or to second progression on the same targeted therapy (some patients received LCT to oligoprogressive sites during TKI therapy in both groups), or death from any cause, according to previously published study [21]. OS was defined from the time of TKI start to the date of death or last follow up. Data represent the median (range) and n (%). Baseline characteristics were compared using the Fisher exact test for categorical variables and the two-sample t-test for continuous variables, as appropriate. Survival was evaluated by Kaplan-Meier plots and the Cox log-rank test, and interactions were tested. Multivariate survival analyses were performed using the Cox proportional hazards regression model. *P*-values < 0.05 were considered to indicate significant differences. The statistical software packages R (http://www.R-project.org, The R Foundation) and Empower Stats (http://www.empowerstats.com, X&Y Solutions, Inc., Boston, MA) were used to analyze all data.

## Results

### Patient characteristics

**Fig. 1:**
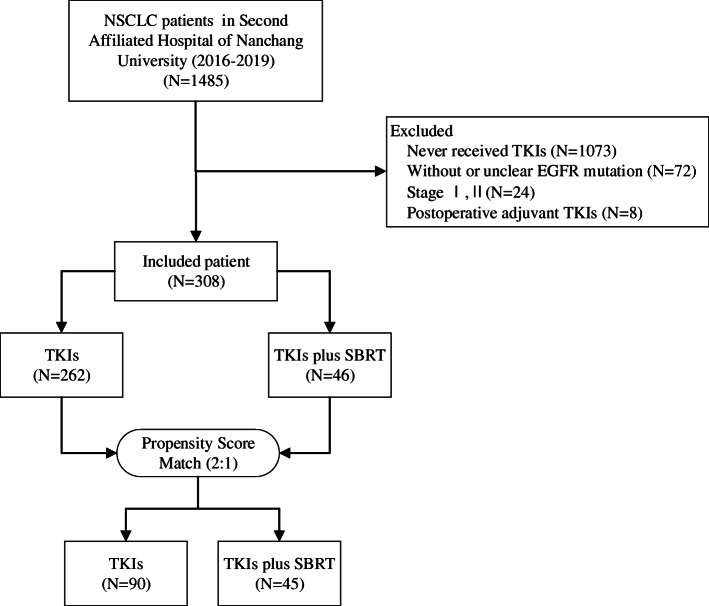
Flow chart of screened patients. NSCLC non-small cell lung cancer, TKIs tyrosine kinase inhibitors, EGFR epidermal growth factor receptor, SBRT stereotactic body radiation therapy

Between August 2016 and August 2019, a total of 1485 patients with NSCLC were hospitalized at our center. Ultimately, 308 patients were included in the study population. Among them, 262 patients received TKIs alone, and 46 patients received TKIs with SBRT. Using PSM with a ratio of 1:2, the study cohort was composed of 45 patients treated with TKIs plus SBRT and 90 patients treated with TKIs only. The flow chart of screened patients is summarized in Fig. 1. Patient and tumor *characteristics are listed in* Table 1. After PSM, there were no significant differences in clinical characteristics between the TKIs and TKIs +SBRT cohorts. The median time on induction TKIs (prior to SBRT) was 9.7 months (95% CI 7.3 m–12.1 m).

**Table 1:** Baseline characteristics of the unmatched and matched groups

Characteristic	Before Propensity Score Matching			After Propensity Score Matching		
	TKIs	TKIs + SBRT	***p*** value	TKIs	TKIs + SBRT	***p*** value
**NO. of patients**	262	46		90	45	
**Median age years (range)**	60.0 (22–87)	59.5 (25–85)	0.843	58.0 (32–83)	59.0 (25–85)	0.614
**Sex**			0.775			0.902
Female	154 (58.8%)	26 (56.5%)		51 (56.7%)	25 (55.6%)	
Male	108 (41.2%)	20 (43.5%)		39 (43.3%)	20 (44.4%)	
**Smoking status**			0.877			0.439
Never	185 (70.6%)	33 (71.7%)		58 (64.4%)	32 (71.1%)	
Former or current	77 (29.4%)	13 (28.3%)		32 (35.6%)	13 (28.9%)	
**ECOG status**			0.462			0.236
0–1	210 (80.2%)	39 (84.8%)		68 (75.6%)	38 (84.4%)	
2–3	52 (19.8%)	7 (15.2%)		22 (24.4%)	7 (15.6%)	
**Prior radical resection**			0.968			0.249
No	233 (88.9%)	41 (89.1%)		73 (81.1%)	40 (88.9%)	
Yes	29 (11.1%)	5 (10.9%)		17 (18.9%)	5 (11.1%)	
**Histological type**			0.482			0.392
Adenocarcinoma	245 (93.5%)	45 (97.8%)		88 (97.8%)	44 (97.8%)	
Adenosquamous carcinoma	8 (3.1%)	0 (0.0%)		1 (1.1%)	0 (0.0%)	
Not otherwise specified	4 (1.5%)	1 (2.2%)		0 (0.0%)	1 (2.2%)	
Squamous carcinoma	5 (1.9%)	0 (0.0%)		1 (1.1%)	0 (0.0%)	
**Clinical stage**			0.372			0.514
III	18 (6.9%)	1 (2.2%)		0 (0.0%)	0 (0.0%)	
IVa	36 (13.7%)	10 (21.7%)		18 (20.0%)	10 (22.2%)	
IVb	179 (68.3%)	30 (65.2%)		55 (61.1%)	30 (66.7%)	
Recurrence	29 (11.1%)	5 (10.9%)		17 (18.9%)	5 (11.1%)	
**Brain metastasis**			0.381			0.143
No	147 (56.1%)	29 (63.0%)		44 (48.9%)	28 (62.2%)	
Yes	115 (43.9%)	17 (37.0%)		46 (51.1%)	17 (37.8%)	
**Metastatic status**			**0.033**			0.43
Polymetastasis	129 (52.9%)	16 (35.6%)		26 (28.9%)	16 (35.6%)	
Oligometastasis	115 (47.1%)	29 (64.4%)		64 (71.1%)	29 (64.4%)	
**EGFR mutations**			0.748			0.787
Exon19 deletion	146 (55.7%)	23 (50.0%)		51 (56.7%)	23 (51.1%)	
L858R mutation	104 (39.7%)	21 (45.7%)		34 (37.8%)	20 (44.4%)	
Other	12 (4.6%)	2 (4.3%)		5 (5.6%)	2 (4.4%)	
**Type of EGFR TKIs**			0.863			0.611
Gefitnib	152 (58.0%)	29 (63.0%)		53 (58.9%)	28 (62.2%)	
Erlotinib	28 (10.7%)	3 (6.5%)		14 (15.6%)	3 (6.7%)	
Osimertinib	26 (9.9%)	5 (10.9%)		10 (11.1%)	5 (11.1%)	
Icotinib	48 (18.3%)	7 (15.2%)		11 (12.2%)	7 (15.6%)	
Afatinib	8 (3.1%)	2 (4.3%)		2 (2.2%)	2 (4.4%)	
**No. of lines of TKIs therapy**			**0.022**			0.361
1	232 (88.5%)	35 (76.1%)		74 (82.2%)	34 (75.6%)	
2	30 (11.5%)	11 (23.9%)		16 (17.8%)	11 (24.4%)	

*TKIs* tyrosine kinase inhibitors, *SBRT* stereotactic body radiation therapy, *ECOG* eastern cooperative oncology group, *EGFR* epidermal growth factor receptor

### Survival outcome

**Fig. 2:**
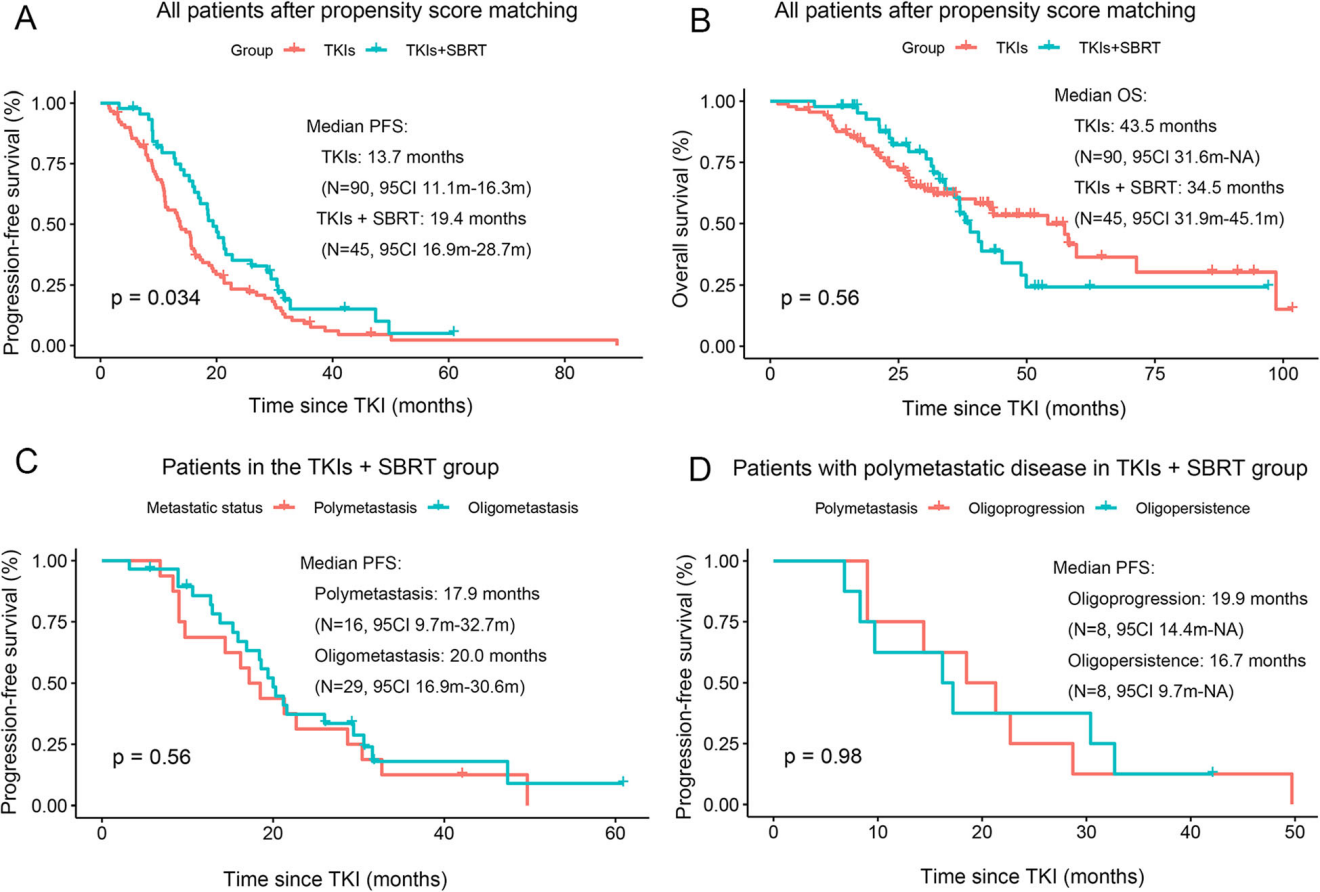
Kaplan–Meier curves for PFS (**a**) and OS (**b**) in patients treated with TKIs + SBRT or TKIs alone; Kaplan-Meier curves of PFS for 45 patients in the TKIs + SBRT group (**c**) and 16 patients with poly-metastatic disease in the TKIs + SBRT group (**d**). PFS progression-free survival, OS overall survival, TKIs tyrosine kinase inhibitors, SBRT stereotactic body radiation therapy, CI confidence interval

**Fig. 3:**
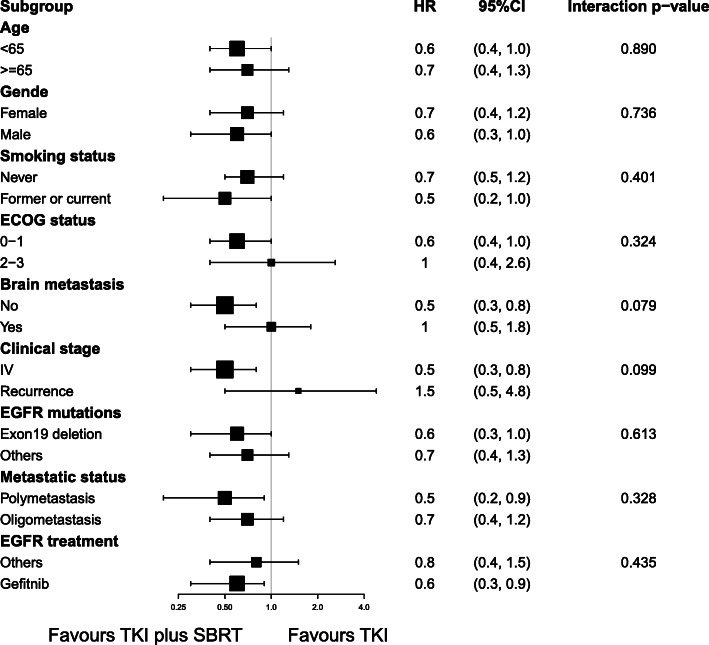
Subgroup analyses of disease-free survival. HR hazard ratio, CI confidence interval, ECOG eastern cooperative oncology group, EGFR epidermal growth factor receptor, TKIs tyrosine kinase inhibitors, SBRT stereotactic body radiation therapy

The median PFS was 19.4 months (95% CI 16.9 m–28.7 m) in the TKIs + SBRT group compared to 13.7 months (95% CI 11.1 m–16.3 m) in the TKIs group, which was significantly different (*p* = 0.034) (Fig. 2a). Comparison of the two groups in all subgroup analyses revealed that patients treated with TKIs plus SBRT were associated with improved PFS compared to patients treated with TKIs (*P*-value> 0.05 for interaction). Hazard ratio (HR), 95% confidence interval (CI) and interaction *p*-value between different subgroups are listed in Fig. 3. Subgroup analysis according to metastatic status in the TKIs + SBRT group showed that median PFS for oligometastatic patients was better compared to polymetastasis patients (20.0 months vs 17.9 months, respectively), but this difference did not achieve statistical significance (*p* = 0.561) (Fig. 2c). For patients with polymetastatic disease in the TKIs + SBRT group (*N* = 16), the median time on induction TKIs was 5.6 months (95% CI 4.0 m–11.9 m) in patients with oligopersistent disease and 10.4 months (95% CI 8.8 m–18.9 m) in patients with oligoprogressive disease. Similarly, there was no significant difference in PFS between the two groups, as shown in Fig. 2d (Log-rank *p* = 0.981). Furthermore, OS was not significantly increased with SBRT (*p* = 0.557) (Fig. 2b). In multivariate analysis, a Cox regression model showed that SBRT was an independent statistically significant positive predictor of better survival, with an HR of 0.617 (*p* = 0.023) (Table 2).

**Table 2:** Multivariate analysis of predictors affecting progression-free survival

Multivariate analysis (***N*** = 135)	HR	95% CI for HR		***p***-value
Variable name				
**Age**	0.992	0.976	1.008	0.309
**Sex**	1.112	0.583	2.121	0.747
**Smoking status**	0.793	0.391	1.612	0.522
**ECOG status**	1.579	0.996	2.504	0.052
**Histology (Adenocarcinoma vs others)**	1.540	0.344	6.894	0.572
**EGFR mutation (19del vs others)**	0.965	0.662	1.407	0.853
**EGFR treatment (Gefitinib vs others)**	1.033	0.699	1.528	0.869
**Metastatic status**	0.709	0.477	1.052	0.088
**Clinical stage (Recurrence vs IV)**	0.665	0.383	1.156	0.148
**Brain metastasis**	0.772	0.512	1.163	0.216
**Use of SBRT**	0.617	0.408	0.935	**0.023**

*HR* Hazard Ratio, *CI* confidence interval, *ECOG* eastern cooperative oncology group, *EGFR* epidermal growth factor receptor, *SBRT* stereotactic body radiation therapy

### Adverse events

Adverse events (AEs) are summarized in Table 3. The addition of thoracic SBRT to TKIs for advanced NSCLC patients with EGFR mutations was well tolerated without severe toxicities. There were no grade 4 to 5 toxicities in either cohort. Rates of grade I/II skin rashes, the most frequent grade I/II AEs, were 41.1% versus 44.4% in the TKIs versus TKIs +SBRT cohorts, respectively (*p* = 0.712). Other common AEs of grade I/II in the TKIs cohort were diarrhea (28.9%), dry skin (28.9%), paronychia (18.9%), mucositis (21.1%), anorexia (14.4%), elevated aminotransferase (25.6%), nausea/vomiting (8.9%), fatigue (7.8%) and interstitial pneumonia (3.3%). Meanwhile, radiation pneumonitis, radiation esophagitis and radiation dermatitis were exclusively observed in patients treated with SBRT. The incidence of fatigue was significantly increased in the TKIs +SBRT group compared to the TKIs group (*p* < 0.001). Grade III/IV AEs rates were comparable between groups, with 9 (20.0%) patients in the TKIs +SBRT cohort and 16 (17.8%) patients in the TKIs cohort (*p* = 0.754). The most common grade III/IV AEs in the TKIs cohort were rash (8.9%), elevated aminotransferase (6.7%) and paronychia (2.2%), while in the TKIs +SBRT cohort, grade III/IV rash and elevated aminotransferase were observed in 6.7 and 8.9% of patients, respectively. Grade III radiation pneumonitis occurred in 2 patients (4.4%) after SBRT.

**Table 3:** Adverse events

Adverse event	Grade I/II (%)			Grade III/IV (%)		
	TKIs (***n*** = 90)	TKIs + SBRT (***n*** = 45)	p	TKIs (***n*** = 90)	TKIs + SBRT (***n*** = 45)	p
**Rash**	37 (41.1%)	20 (44.4%)	0.712	8 (8.9%)	3 (6.7%)	0.656
**Diarrhea**	26 (28.9%)	14 (31.1%)	0.790	0 (0.0%)	0 (0.0%)	
**Dry skin**	26 (28.9%)	11 (24.4%)	0.585	0 (0.0%)	0 (0.0%)	
**Paronychia**	17 (18.9%)	8 (17.8%)	0.876	2 (2.2%)	0 (0.0%)	0.552
**Mucositis**	19 (21.1%)	9 (20.0%)	0.881	0 (0.0%)	0 (0.0%)	
**Anorexia**	13 (14.4%)	6 (13.3%)	0.861	0 (0.0%)	0 (0.0%)	
**Elevated aminotransferase**	23 (25.6%)	14 (31.1%)	0.495	6 (6.7%)	4 (8.9%)	0.642
**Nausea or vomiting**	8 (8.9%)	6 (13.3%)	0.425	0 (0.0%)	0 (0.0%)	
**Interstitial pneumonia**	3 (3.3%)	1 (2.2%)	0.858	0 (0.0%)	0 (0.0%)	
**Radiation pneumonitis**	0 (0.0%)	15 (33.3%)	**< 0.001**	0 (0.0%)	2 (4.4%)	0.109
**Radiation esophagitis**	0 (0.0%)	3 (6.7%)	**0.035**	0 (0.0%)	0 (0.0%)	
**Fatigue**	7 (7.8%)	14 (31.1%)	**< 0.001**	0 (0.0%)	0 (0.0%)	
**Radiation dermatitis**	0 (0.0%)	5 (11.1%)	**0.004**	0 (0.0%)	0 (0.0%)	

*SBRT* stereotactic body radiation therapy, *TKIs* tyrosine kinase inhibitors

### Mechanisms of acquired resistance

**Fig. 4:**
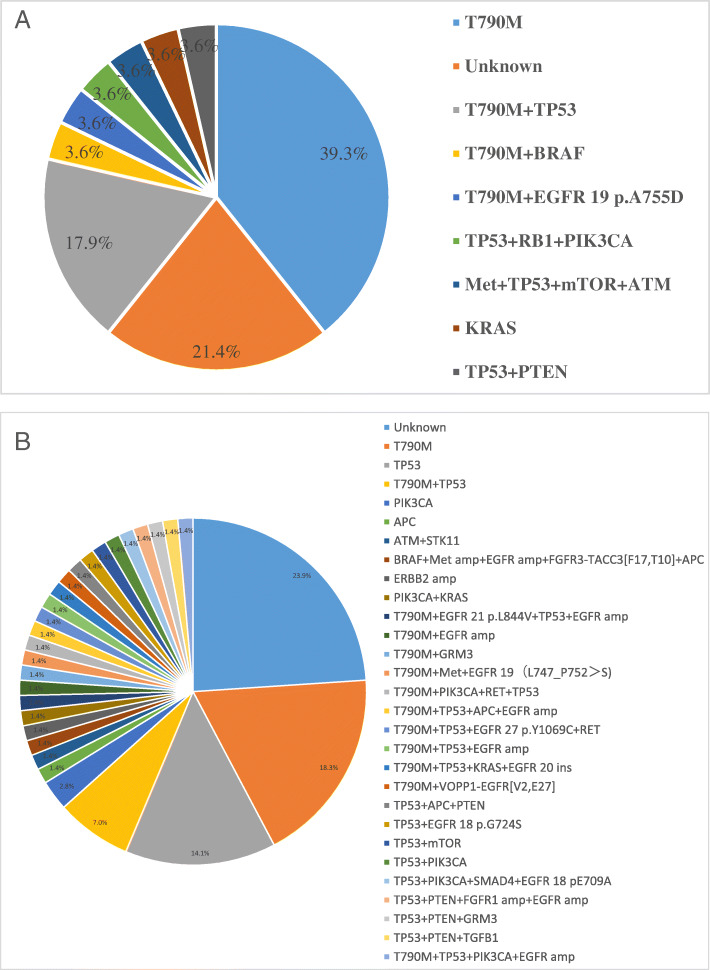
Resistance mechanisms clarified by patient in TKIs+SBRT (**a**) and single TKIs (**b**) groups (Gefitinib, Erlotinib, Afatinib and Icotinib). SBRT stereotactic body radiation therapy, TKIs tyrosine kinase inhibitors

Of the 135 patients evaluated, 99 (73%) had plasma cfDNA NGS performed at baseline and disease progression on first-generation or second-generation TKIs until September 2020. Mechanisms of acquired resistance to either TKIs + SBRT or single TKIs are shown in Fig. 4(a-b). The cumulative calculation for patients with treatment-emergent oncogenic alterations at disease progression in the TKIs +SBRT and single TKIs groups are shown in Table 4. In the TKIs +SBRT group, NGS results showed that T790M mutations were detected in 64.3% (18/28) of patients, followed by TP53 mutations in 28.6% (8/28), BRAF mutations in 3.6% (1/28), ATM mutations in 3.6% (1/28), Met amplification in 3.6% (1/28), mTOR mutation in 3.6% (1/28), KRAS mutations in 3.6% (1/28), PTEN mutations in 3.6% (1/28), EGFR 19 p.A755D mutations in 3.6% (1/28), RB1 mutations in 3.6% (1/28) and PIK3CA mutations in 3.6% (1/28). Approximately 78.6% (22/28) of patients in the TKIs +SBRT group had known causes of drug resistance. In addition, 21.40% of patients exhibited only the original EGFR sensitive mutation. In contrast, in the TKIs cohort, although T790M was also the predominant acquired resistance mechanism, patients in the TKIs cohort exhibited fewer T790M-positive mutations (40.8%, *p* = 0.035) compared to patients in the TKIs +SBRT cohort. Other acquired mutations in the TKIs group are shown in Table 4.

**Table 4:** The cumulative calculation for patients with treatment-emergent oncogenic alterations

Gene alterations	TKIs (***n*** = 71)	TKIs + SBRT (***n*** = 28)	***P***-value
**TP53**	30 (42.3%)	8 (28.6%)	0.207
**T790M**	29 (40.8%)	18 (64.3%)	**0.035**
**Unknown**	17 (23.9%)	6 (21.4%)	0.790
**PIK3CA**	7 (9.9%)	1 (3.6%)	0.435
**EGFR amp**	7 (9.9%)	0 (0.0%)	0.186
**PTEN**	4 (5.6%)	1 (3.6%)	1
**APC**	4 (5.6%)	0 (0.0%)	0.575
**Met amp**	2 (2.8%)	1 (3.6%)	1
**KRAS**	2 (2.8%)	1 (3.6%)	1
**GRM3**	2 (2.8%)	0 (0.0%)	1
**RET**	2 (2.8%)	0 (0.0%)	1
**BRAF**	1 (1.4%)	1 (3.6%)	0.488
**ATM**	1 (1.4%)	1 (3.6%)	0.492
**mTOR**	1 (1.4%)	1 (3.6%)	0.488
**FGFR3-TACC3[F17,T10]**	1 (1.4%)	0 (0.0%)	1
**EGFR18 pE709A**	1 (1.4%)	0 (0.0%)	1
**SMAD4**	1 (1.4%)	0 (0.0%)	1
**ERBB2 amp**	1 (1.4%)	0 (0.0%)	1
**FGFR1 amp**	1 (1.4%)	0 (0.0%)	1
**EGFR 21 p.L844V**	1 (1.4%)	0 (0.0%)	1
**EGFR 20 ins**	1 (1.4%)	0 (0.0%)	1
**EGFR 19(L747_P752>S)**	1 (1.4%)	0 (0.0%)	1
**EGFR 27 p.Y1069C**	1 (1.4%)	0 (0.0%)	1
**EGFR 18 p.G724S**	1 (1.4%)	0 (0.0%)	1
**TGF β1**	1 (1.4%)	0 (0.0%)	1
**VOPP1-EGFR [V2,E27]**	1 (1.4%)	0 (0.0%)	1
**STK11**	1 (1.4%)	0 (0.0%)	1
**EGFR 19 p.A755D**	0 (0.0%)	1 (3.6%)	0.283
**RB1**	0 (0.0%)	1 (3.6%)	0.283

*SBRT* stereotactic body radiation therapy, *TKIs* tyrosine kinase inhibitors

## Discussion

Evidence from the literature on patients with EGFR-mutated NSCLC indicates that disease progression after TKIs occurs most often at sites of disease known to exist at baseline, supporting the idea of disease progression due to the development of TKI-resistant clones at the primary tumor site with subsequent systemic reseeding and widespread distant progression [14, 22–24]. Recently, due to advancements in radiotherapy, SBRT has allowed for delivery of high precision and dose escalated treatment to targets throughout the body and has been commonly used in selected patients with and without metastatic lesions, with excellent rates of local control and acceptable toxicity [25–28]. The potential advantages of preemptive LCT to residual tumors after targeted therapy in nonprogressing patients, and the use of SBRT for oligoprogressive sites, are that it may delay or prevent the emergence of resistant clones before additional metastatic spread occurs, as suggested by the observation that LCT delays the time to new metastases [11, 12, 21, 22, 29].

Nevertheless, many unanswered questions remain regarding optimal timing of SBRT, selection of primary and metastatic locations for SBRT, and the optimal patient population (oligometastic versus polymetastatic disease). Our study is one of the few trials of real-world data to compare the efficacy and safety of TKIs plus SBRT to TKIs alone in EGFR-mutant NSCLC and the first report of acquired potential resistance mechanisms to TKIs plus thoracic SBRT using a large panel of NGS tests.

In the present study, we retrospectively reviewed the clinical database of our center and found that patients who underwent TKIs plus SBRT exhibited longer PFS compared to patients treated with TKIs alone (19.4 vs 13.7 months, respectively, Log-rank *p* = 0.034). However, an influence on OS has not yet been shown (*p* = 0.557). OS data are immature at present, which may be the possible explanation for this result. At data cutoff, 41 deaths (45.5%) had occurred in the TKIs group and 23 events (51.1%) in the TKIs +SBRT group. Long follow-up may be required for reliable and further evaluations. Several previous retrospective and clinical randomized controlled studies of patients with EGFR mutant NSCLC following EGFR TKIs therapy demonstrated that local therapy with surgery or radiation may lead to increased PFS [11, 12, 30]. There were several important weaknesses in those studies. First, they were single arm studies with a small number of patients [11, 30], and only 10 and 16 patients were treated with SBRT, respectively. Second, there were differences in the baseline characteristics compared to the local therapy group and screen failure group [11, 12, 30]. Our retrospective study has a relatively larger sample size. Additionally, adoption of PSM balances baseline patient characteristics between groups and minimizes some weaknesses of a retrospective study. Our conclusions are generally consistent with those of the three abovementioned studies. However, the PFS of TKIs plus SBRT in our study was 19.4 months, which was shorter than that reported in the previous study (36 months) [12]. Potential reasons for this discrepancy are as follows: patients in the present study only underwent SBRT on lung lesions, and the inclusion criteria are closer to real-world circumstances. Though the results were encouraging, the current study has several important limitations, including a relatively small sample size and retrospective analysis of potential hidden biases performed at a single center.

Targetable mutations have a key role in identifying treatment options in NSCLC. The most common mechanisms of acquired resistance can be classified as three types: target gene modification, bypass of signaling pathway activation and histological or phenotypic transformation [31]. Several mechanisms of resistance have been identified, of which the T790M mutation is most prevalent. T790M was reported in approximately 50% of cases whose disease progressed on a first-generation or second-generation TKIs [32–35]. Considering the heterogeneity of tumor tissue samples and the convenience of fluid biopsy [24, 36–38], mutation status was assessed and analyzed in liquid biopsy samples by NGS in our study. We observed similar frequencies of T790M mutation in the TKIs group. However, the frequency of T790M mutation in the TKIs plus SBRT group was significantly higher compared to the TKIs alone group (*p* = 0.035). The cumulative incidence of T790M frequencies suggests the possibility that SBRT plus TKIs might increase the emergence of this resistance mechanism. This higher frequency of detection of T790M might be due to the presence of intratumoral heterogeneity and the elimination of subclones by the selection pressures from SBRT. It was reported in a preclinical study that gefitinib treatment increases clonogenic cell killing by radiation but only in cell lines sensitive to gefitinib alone [39]. Another possible explanation is that the concentration of cfDNA is limited and radiotherapy can increase tumoral cfDNA levels in the plasma for NGS. One published series investigating the impact of radiotherapy on cfDNA in NSCLC demonstrated that radiotherapy increases tumoral cfDNA levels in the plasma and showed that T790M mutation in a case cfDNA sample was detected only after radiotherapy [40]. In esophageal squamous cell carcinoma, Ru et al. [41] found several mutations (STK11, KRAS and APC) were detected in the post-radiation samples, whereas these were not present in the baseline samples. We have collected serial blood samples and would like to conduct further basic study to verify the role of SBRT in T790M. Because of the low mutation rate and the limitation of sample size, other treatment-emergent oncogenic alteration frequencies were similar between groups. After acquiring resistance to EGFR-TKIs therapy, it is important to identify the definitive mechanisms of acquired resistance in all patients explored. Then, based on genotyping, depending on the existence of the T790M mutation or other oncogenic alterations, subsequent treatment can be chosen, according to current NSCLC guidelines [31, 42, 43].

## Conclusion

In this retrospective study, TKIs plus SBRT conveyed superior PFS versus TKIs in advanced EGFR-mutated NSCLC with acceptable toxicity in clinical practice. Moreover, the frequency of the EGFR T790M mutation seemed to be increased in patients treated with additional thoracic SBRT. Distinct mechanisms should be assessed in disease progression on TKIs, which is a crucial step in guiding future treatment. Prospective and randomized trials will be required to validate and expand the findings of our study.

## Data Availability

The datasets used and/or analyzed during the current study are available from the corresponding author on reasonable request.

## Abbreviations

EGFR: Epidermal growth factor receptor
TKIs: Tyrosine kinase inhibitors
SBRT: Stereotactic body radiation therapy
PSM: Propensity score matching
cfDNA: Cell-free DNA
NSCLC: Non–small-cell lung cancer
OS: Overall survival
PFS: Progression-free survival
NGS: Next-generation sequencing
LCT: Local consolidation therapy
HR: Hazard ratio
CI: Confidence interval
AEs: Adverse events
ECOG: Eastern cooperative oncology group
